# The first-generation anatomical medial meniscus prosthesis led to unsatisfactory results: a first-in-human study

**DOI:** 10.1007/s00167-022-07205-x

**Published:** 2022-11-07

**Authors:** T. G. van Tienen, B. van Minnen, K. C. Defoort, P. J. Emans, S. A. W. van de Groes, N. Verdonschot, L. M. Jutten, R. W. E. Pikaart, P. J. C. Heesterbeek

**Affiliations:** 1 Radboud University Medical Centre, Geert Grooteplein Zuid 10, 6525 GA Nijmegen, The Netherlands; 2 ATRO Medical BV, Liessentstraat 9-A, 5405 AH Uden, The Netherlands; 3 Sint Maartenskliniek Nijmegen, Hengstdal 3, 6574 NA Ubbergen, The Netherlands; 4 Joint-Preserving Clinic, Department of Orthopaedic Surgery, Maastricht University Medical Centre, P. Debyelaan 25, 6229 HX Maastricht, The Netherlands

**Keywords:** Meniscectomy, Meniscus replacement, Artificial meniscus, Meniscus prosthesis, Post-meniscectomy pain syndrome

## Abstract

**Purpose:**

The purpose of this first-in-human study was to evaluate the effect of a polycarbonate anatomical meniscus prosthesis system, including the surgical procedure, on knee pain and describe potential adverse events in patients with post-meniscectomy pain syndrome.

**Methods:**

Eleven patients with post-meniscectomy pain syndrome and limited underlying cartilage damage were enrolled in the study. Five received a medial polycarbonate urethane meniscus prosthesis which was clicked onto 2 titanium screws fixated at the native horn attachments on the tibia. The KOOS score was planned to be collected at baseline and at 3, 6, 12 and 24 months following the intervention including radiographs at 6, 12 and 24 months. MRI scans were repeated after 12 and 24 months.

**Results:**

The surgical technique to select an appropriately sized implant and correct positioning of the fixation screws and meniscus prosthesis onto the tibia was demonstrated to be feasible and reproducible. Inclusion stopped after 5 patients because of serious adverse device-related events. All patients reported knee joint stiffness and slight effusion in their knee at 6 months follow-up. In 3 patients the implant was removed because of implant failure and in 1 patient the implant was removed because of persistent pain and extension limitation. In none of the patients did the KOOS score improve in the first 6 months after surgery. However, in the patient who still has the implant in situ, PROMs started to improve 1 year after surgery and this improvement continued through 2 years of follow-up. The KOOS Pain, symptoms and ADL were close to the maximal 100 points. KOOS QoL and sport did improve but remained suboptimal.

**Conclusion:**

This first version of the meniscus prosthesis led to impaired knee function and failed in four out of five patients. The patients where the prosthesis was removed were salvable and the PROMs returned to pre-study levels. The results in the patient where the device is still in place are promising.

**Level of evidence:**

Level II.

## Introduction

Currently, for symptomatic post (sub)total meniscectomy patients with stable and well-aligned knees (‘post-meniscectomy pain syndrome’), a meniscal allograft transplantation is the only treatment option and has been proven to relieve pain and improve knee function [20, 21]. However, post-implantation remodelling and shrinkage of the graft tissue potentially affect its functioning [7, 9]. There is also a shortage of meniscus allografts. As a result of this scarceness in meniscal allografts, transplantation is preserved for patients younger than 50 years of age [3], while studies show the older population also could benefit from this procedure [16].

An artificial meniscus prosthesis can potentially overcome the above-mentioned disadvantages of meniscal allografts and address a clinical need in a broad patient population. Various research groups have attempted to develop prostheses that bear the right characteristics to take over the crucial functions of the native meniscus. However, only a small amount reached the market [8]. Only one synthetic non-resorbable implant, i.e. a free-floating polycarbonate polyurethane (PCU) disc-shaped implant, has been approved for clinical use in Europe and favourable results of a US clinical trial were published recently [12]. This fibre-reinforced PCU disc requires a peripheral rim of the native meniscus to be present [2] to reduce the chance of dislocation and therefore may not provide a suitable solution for total meniscectomy patients. That the implants must survive in a hostile environment is clear given the considerable amount of revisions in their clinical studies [2, 12].

Considering the beneficial effect of the meniscus allograft there may be a need for an artificial meniscus prosthesis that acts like an allograft meniscus.

Since 2008 a meniscus prosthesis has been developed as an alternative for the meniscus allograft. The prosthesis is based on mechanical [13, 18], finite element modelling [6], tribology [11] and animal studies [19]. These studies led to an anatomical meniscus prosthesis for the medial compartment of the knee. The hypothesis was that this meniscus prosthesis is safe in use and will decrease pain in the knee. The purpose of this first-in-human study was to evaluate the safety, feasibility and effect of the anatomical meniscus prosthesis system, including the surgical procedure, on the pain and describe potential adverse events in patients with post-meniscectomy pain syndrome.

## Material and methods

This study was set up as a first-in-human, prospective, multi-centre, open-label, single-arm clinical investigation and approved for 18 patients by the local medical ethical review board (METC Arnhem-Nijmegen number: 2017–3988) and registered in the Dutch Trial Register (NTR: NL75393.000.21). A written informed consent was obtained for each patient in the study.

### Patient population

**Fig. 1:**
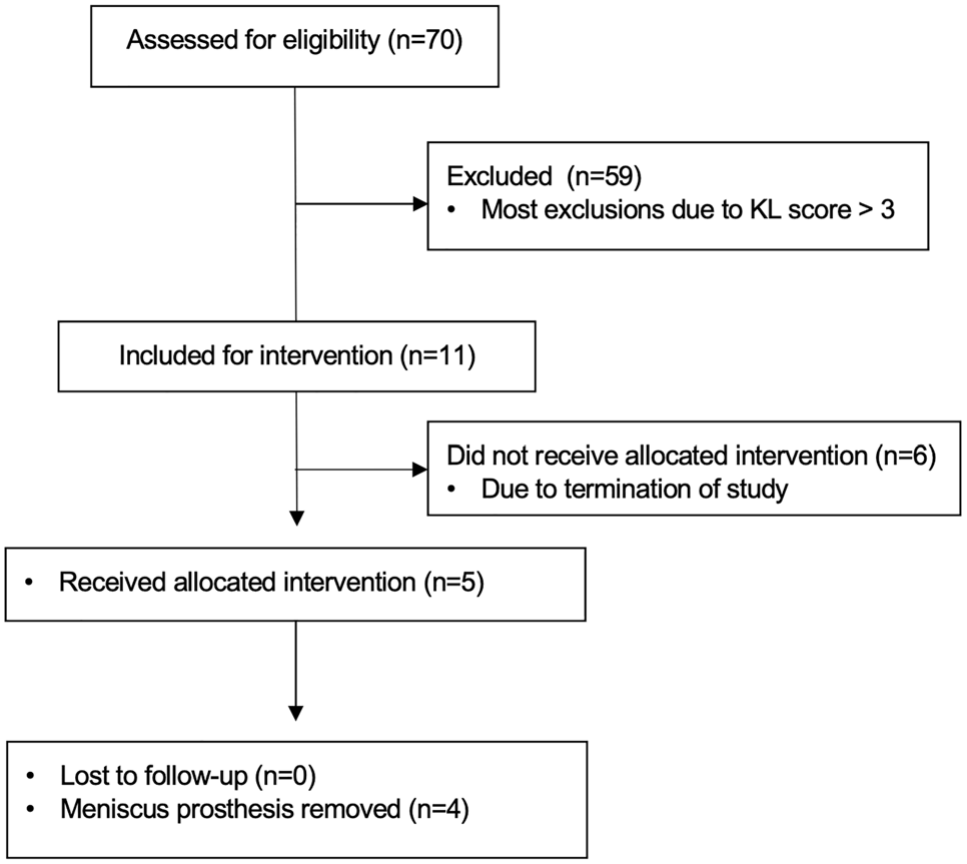
Patient screening flowchart. Of the planned 18 patients, only 11 were included at the moment of termination of the study

Three Dutch hospitals were involved in the study (Radboud University Medical Centre, Nijmegen (RUMC), Sint Maartenskliniek Nijmegen (SMK) and Maastricht University Medical Centre (MUMC)). In Fig. 1 the screening flow diagram is shown. In total, 5 patients underwent a medial meniscus replacement with an artificial meniscus prosthesis between February and September 2019.

Surgeries were performed by 2 experienced knee surgeons (KD, PE). The most important inclusion criteria and exclusion criteria are listed in Table 1. The entire list with inclusion and extrusion criteria is published on www.toetsingonline.nl (NL64121.091.17).

**Table 1:** Inclusion and exclusion criteria

Inclusion Criteria 1. Has medial compartment joint pain with a medial partial meniscectomy > 6 months ago as confirmed by patient history and MRI 2. Has a KOOS Pain of ≤ 75 (100 being the highest attainable and 0 being no pain) 3. Is between age 30 and 65 years (inclusive) at the time of screening 4. Has neutral alignment ± 5º of the mechanical axis, i.e., the angle formed by a line drawn from the centre of the femoral head to the medial tibial spine and a line drawn from the medial tibial spine to the centre of the ankle joint, as confirmed by Radiographs 5. Is willing to be implanted with the meniscus prosthesis 6. Is able to do the study's required follow-up visits, questionnaires, radiographs, CT-scans, and MRIs 7. Is able and willing to understand and sign the study's Informed Consent Form 8. Is able to read and understand the national language of the country in which the relevant clinical site is located	Exclusion criteria 1. Has a symptomatic knee because of a tear that could be addressed by a repeat partial meniscectomy 2. Has evidence of a modified Outerbridge Grade IV cartilage loss on the medial tibial plateau or femoral condyle that potentially could contact a meniscus prosthesis (e.g., a focal lesion > 0.5 cm correlating to a circular defect of > 8 mm in diameter) 3. Has lateral compartment pain and Grade III or Grade IV modified Outerbridge cartilage score in the lateral compartment 4. Has a varus alignment that is not passively correctable 5. Has a laxity level of more than Grade II (IKDC), primary or secondary to an injury of the anterior cruciate ligament (ACL) and/or posterior cruciate ligament (PCL) and/or lateral collateral ligament (LCL) and/or medial collateral ligament (MCL) 6. Compared to a normal knee, has obvious radiological evidence of medial femoral squaring, anatomical variance in the medial tibial plateau or irregularly shaped cartilage surface 7. Had an ACL reconstruction performed < 9 months prior to surgery 8. Has a BMI > 32.5 at the time of screening

### Artificial meniscus prosthesis

**Fig. 2:**
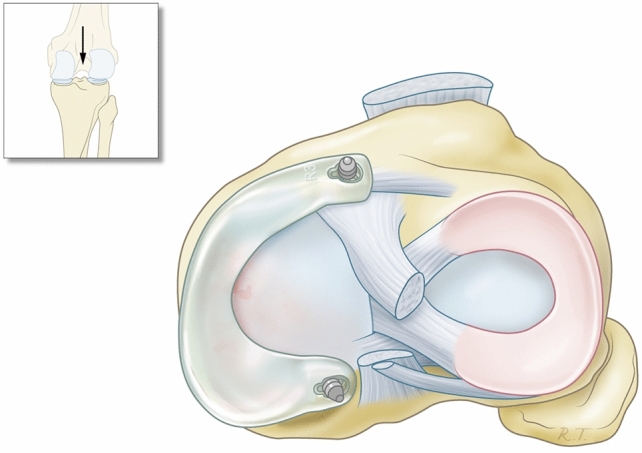
Artificial medial meniscus prosthesis

**Fig. 3:**
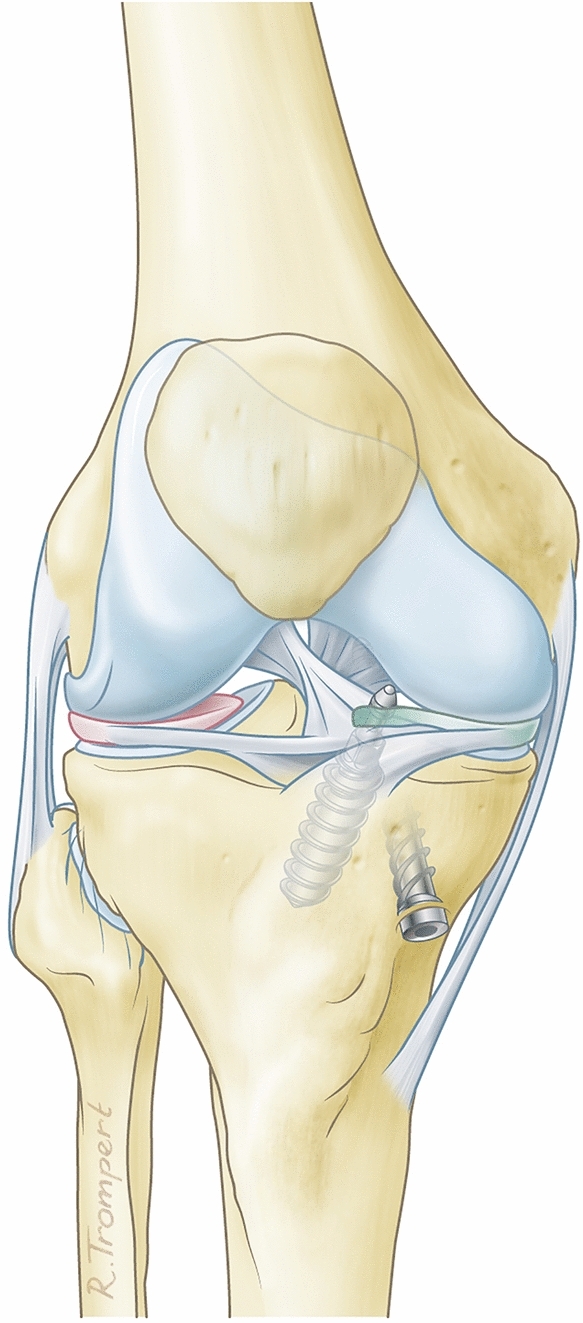
Artificial meniscus prosthesis with screws for fixation in the tibia

The synthetic polymer meniscal implant (ATRO Medical BV, Nijmegen, The Netherlands) (Fig. 2) was developed to treat patients with a painful medial knee compartment with a deficient meniscus. A statistical shape model was created, containing the meniscus geometries of 35 subjects (20 females, 15 males) that were obtained from MR images [17]. Based on this model an average geometry meniscus prosthesis could be composed and based on a finite element model [6]. Five different sizes for both the left and right knee (L1–5, R1–5) could be injection-moulded. Medical-grade PCU was used for the meniscus prosthesis, allowing a flexible two-component construct with a softer variant PCU in contact with the cartilage and a stiffer reinforcement ring in the core of the implant to resist the circumferential forces and mimic the circumferential strength of the native meniscus. However, this reinforcement ring made the prosthesis significantly stiffer in the horizontal plane on the tibial plateau than the native meniscus. This means that the prosthesis may move less easily with the femoral condyle than the native meniscus. The meniscus prosthesis is attached to the tibia plateau solely by an anterior and posterior horn fixation. The prosthesis is clicked onto 2 titanium screws, specifically designed for this procedure. The screws are placed in the tibial plateau at the native meniscus horn location. The prosthesis requires no peripheral fixation to the capsule. In case the prosthesis has to be removed, only two 3.2 mm drill holes remain, which quickly close again (Fig. 3).

### Surgical technique

After anaesthesia, knee stability was evaluated by the surgeon to confirm a stable knee joint and to assess the preoperative range of motion of the knee joint. The procedure started with a standard arthroscopy and the indication for the meniscus prosthesis was confirmed. When the indication was confirmed, the remnants of the medial meniscus were removed. The medial parapatellar vertical incision was extended to approximately 5 cm in length. An aiming device and trial sizers were designed specifically for this procedure. With the trial sizers, the size of the meniscus prothesis was determined based on the following landmarks: the eyelets of the trial sizer should be located on the anatomic meniscus horn attachments and the contour of the trial sizer should follow the tibial plateau edge.

A 3.2 mm Steinmann pin was drilled upwards under arthroscopic control and emerged through the posterior eyelet of the trial sizer, exactly on the posterior anatomic location of the horn of the native meniscus. The screw hole was tapped and the specially designed posterior fixation screw was placed until the neck of the screw was fully visible in the back of the knee. For placement of the anterior screw, the trial sizer was left in place with the eyelet around the posterior screw. The exact location of the anterior drill hole was determined by rotating the trial sizer around the posterior screw. The specially designed anterior screw was placed. Subsequently, the meniscus prosthesis was placed above the screw heads and the posterior horn of the meniscus prosthesis was pushed onto the screw head and followed by the anterior horn of the prosthesis.

### Rehabilitation

The rehabilitation protocol was developed specifically for this procedure and started with 4 weeks of partial weight bearing (50%) with mobilization on crutches and increasing the load based on pain and swelling. No brace or immobilization was applied. This partial weight bearing was required to allow the implant to become saturated with water (~ 1%) before full loading to ensure that the maximum flexibility of the prosthesis is achieved. Because the prosthesis is somewhat stiffer in the plane of the tibial plateau and therefore less mobile during flexion and extension, the material must additionally adapt by creep. This creep occurs when the patient puts weight on the implant [15]. When the knee joint achieved a full range of motion, all restrictions were abandoned, except the advice to avoid high-impact activities and sports. Given the early stage of development of this new meniscus prosthesis, it is not yet known how long the implant can withstand these high loads while practising sports.

### Follow-up

Primary outcome for this preliminary analysis was Knee injury and Osteoarthritis Outcome Score (KOOS) Pain, assessed at baseline, 3, 6, 12 and 24 months. In addition, KOOS symptoms, Activities of Daily Living (ADL), Sports and Recreation, Quality of Life (QoL), the Lysholm score and the IKDC score were assessed. Weightbearing radiographs on the preoperative screening were taken and at 12 and 24 months to evaluate screw position and fixation. MRI scans (metal artifact reduction sequence) were repeated after 12 and 24 months to evaluate implant integrity and to evaluate the status of the cartilage via the modified Outerbridge grading scale. Other secondary outcomes also included adverse device effects (ADEs) during 24 months of follow-up. Due to implant removal in four out of five patients not all data could be collected.

### Statistical analysis

The primary endpoint was the performance of the meniscus prosthesis, measured as a decrease in the KOOS Pain subscale. The minimal clinically important change (MIC) of the KOOS is 8–10 points [14]. When an MIC of 8 is applied calculating the sample size (mean difference) and a standard deviation of 10 points (based on pain improvement of an alternative meniscus prosthesis) [12], the size of the group should be 15 patients. To compensate for a dropout percentage of 20% (loss to follow-up, incompleteness of data, complications with surgery or rehabilitation), the sample size was set at 18.

All (available) patients were followed over time and the patient-related outcome measures (PROMs) and other outcomes are presented with descriptive statistics such as median and interquartile ranges.

## Results

### Patients

In total, 11 patients were included for intervention, which is approximately 10% of the screened patients, and qualified to be enrolled for operation. Of those, 5 patients received the meniscal implant, 4 females and 1 male. In 4 patients, a size 2 was implanted and in 1 patient a size 3 was used. Two patients had a grade 3 modified Outerbridge score on the femur, 2 had grade 2 and 1 had grade 1. On average, 3 years had elapsed since patients’ last (partial) meniscectomy. The other enrolled patients did not undergo surgery because the study was prematurely terminated due to a number of device-related serious adverse events during the trial. Baseline characteristics are shown in Table 2.

**Table 2:** Patient characteristics

Patient characteristic	Median (Q1–Q3)
Age	47 (45–51)
BMI	24.4 (22.7–28)

In 4 patients the meniscus prosthesis was removed. During explantation, the cartilage state was unchanged compared to the preoperative state and had not deteriorated. After explantation, the PROMs returned to pre-study levels. Two patients received an unicondylar knee arthroplasty several months after the explantation of the meniscus prosthesis.

### Patient outcomes

Patients were admitted to the hospital for 24 h and received physiotherapy. As soon as the patients were able to walk on crutches without weight bearing and were comfortable with the pain, they were discharged.

**Fig. 4:**
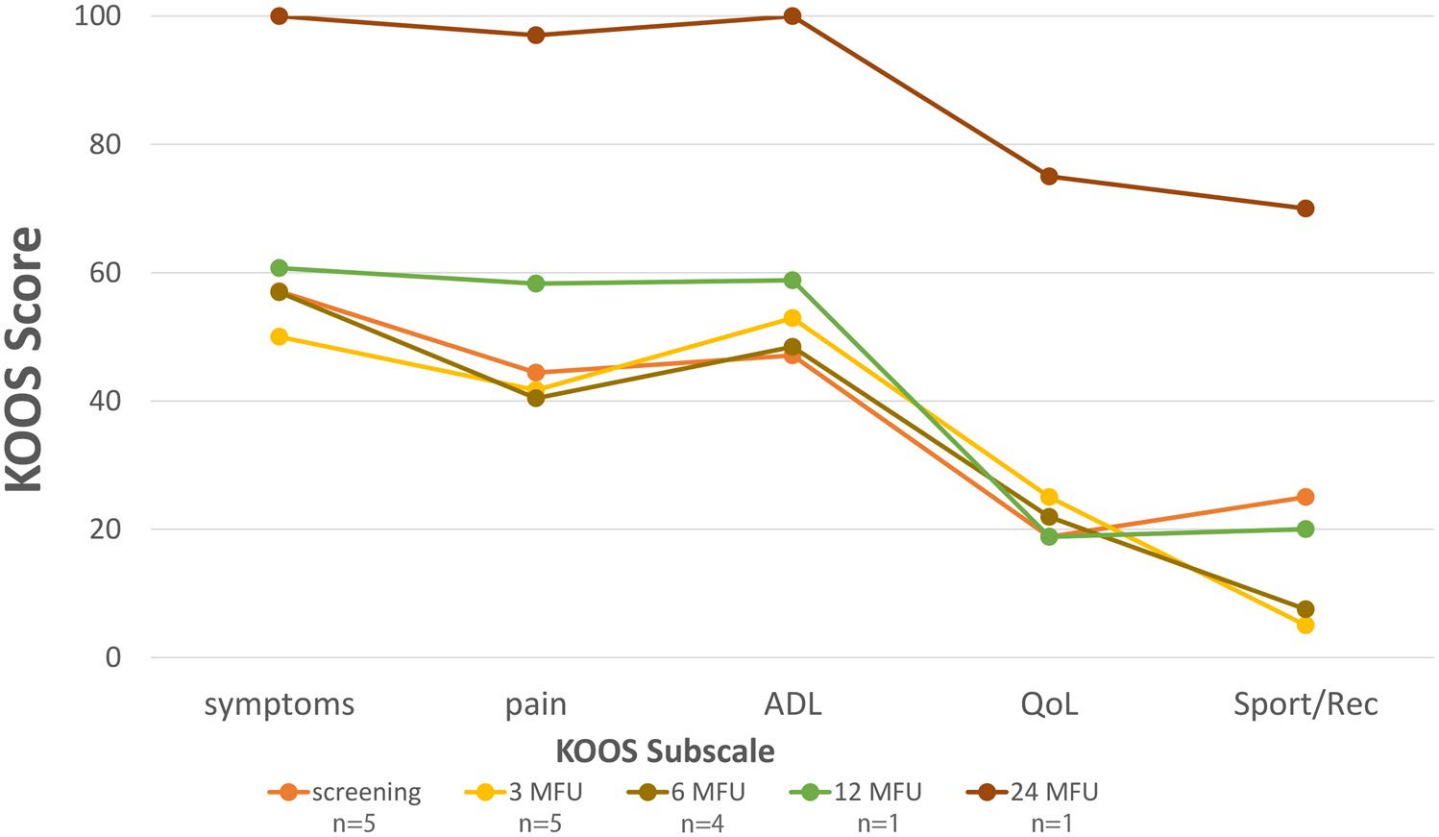
Median KOOS scores

**Fig. 5:**
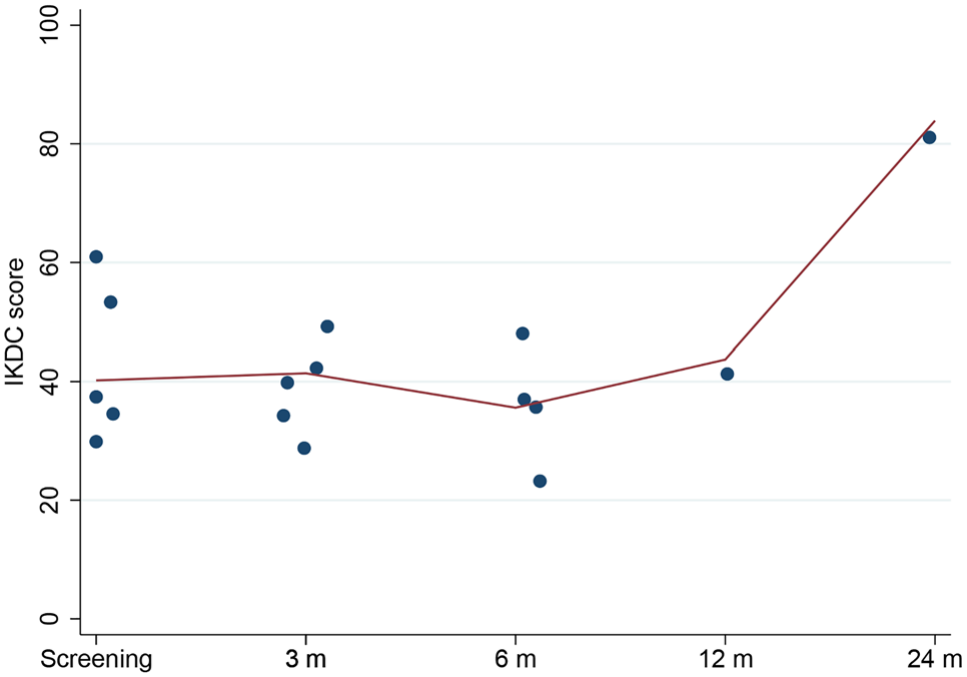
IKDC score. Dots represent individual scores, solid line represents the median score. Note: at 12 and 24 months follow-up *n* = 1

**Fig. 6:**
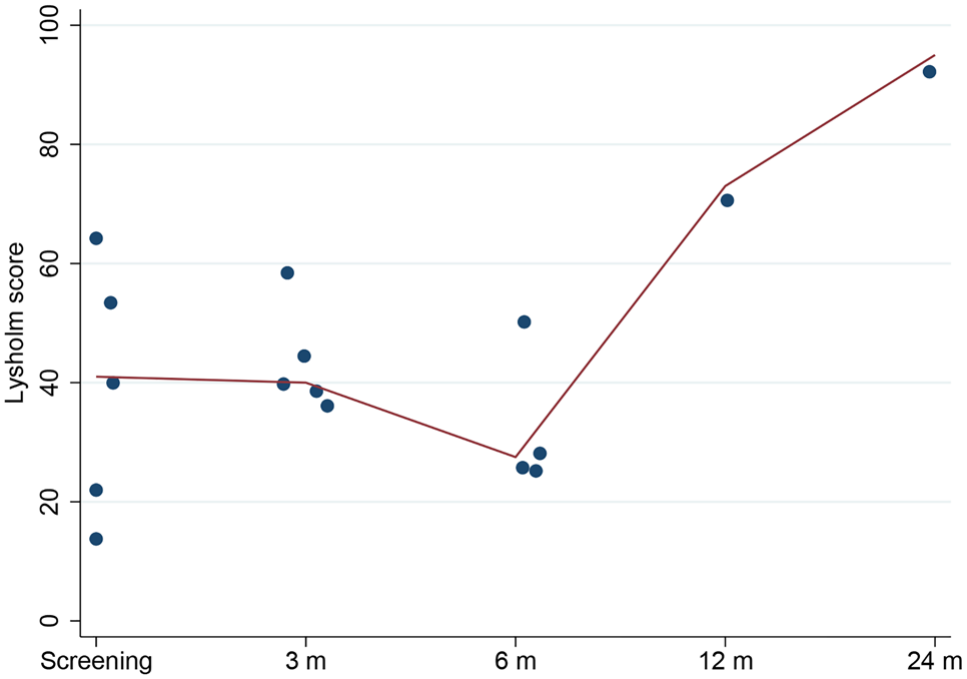
Lysholm score. Dots represent individual scores, solid line represents the median score. Note: at 12 and 24 months follow-up *n* = 1

Rehabilitation after surgery was slower than expected. Six weeks after surgery, the knees remained slightly swollen and irritated. As a result, the patients also walked longer with crutches than the pre-specified 4 weeks. The PROMs remained low and did not improve from baseline scores for the first 6 months (Figs. 4, 5, 6). One year after surgery, there was only 1 patient in the study. In all other patients, the implant had been removed. In this patient with the implant in situ, the PROMs seemed to improve at 12 months follow-up. After 2 years the knee was nearly pain-free with almost maximum scores in KOOS symptoms (100), pain (97) and ADL (100), and the Lysholm (95) and IKDC (83.9) also showed nearly optimal scores (Figs. 4, 5, 6). Radiographs showed no loosening of the fixation screws or a changed position.

### MRI cartilage grading systems

Only 1 patient with the implant in situ underwent the planned 1- and 2-year MRI scans. At the screening visit, the cartilage in the femur and tibia was grade 2 on the modified Outerbridge grading scale. At 24-month follow-up the cartilage status had not changed in this patient.

### Adverse events

Table 3 describes the adverse events that occurred.

**Table 3:** Adverse events

Patient	Adverse event 1	Time after surgery of AE documentation (months)	First Intervention	Adverse event 2	Implant failure on MRI	Withdrawal from study (months after surgery)
1 201	Prolonged knee joint stiffness and pain	3	MUA^a^		No	Yes, after implant removal (7 months)
2 203	Prolonged knee joint stiffness and pain	3	MUA		Yes (posterior horn tear)	Yes, after implant removal (7 months)
3 302	Prolonged knee joint stiffness and pain	4			Yes (small parrot beak tear mid horn (wear))	Yes, after implant removal (6 months)
5 101	Prolonged knee joint stiffness and pain	3		Patient had an acute moment and thereafter pain and swelling knee	Yes (fixation hole tear)	Yes, after implant removal (8 months)

^a^MUA: manipulation under anaesthesia

All patients reported a stiffer knee with slight effusion with approximately 10° of flexion deficit and 5° of extension deficit at their latest follow-up. In four out of five patients this was documented as an adverse event at 3 months after surgery. Due to the flexion contracture 3 months after surgery, it was decided to perform a manipulation under anaesthesia (MUA) in patients 1 and 2 in an attempt to increase the flexion. This MUA did not increase flexion but patient 2 reported that the pain had increased after the MUA. An additional MRI revealed a torn implant at the posterior horn and the implant was removed. Because of this adverse event, all patients were scheduled for an extra MRI of their knee joint. In patient 1 the implant was still intact but was removed due to an impinging anterior horn resulting in persistent pain and extension deficit. Patient 3 showed a damaged implant surface with a small tear due to contact with a small area of bare bone. Because the knee was still stiff and painful the meniscus prosthesis was removed. Patient 5 had tripped with a deep flexion event in the knee and the implant was removed after MRI confirmation that the implant was torn at the posterior fixation ring. The state of the cartilage in the medial compartment on these MRI scans was not different compared with preoperative scans.

## Discussion

The hypothesis was that the implant was safe and would reduce knee joint pain in the patients. This was not supported by the results and the hypothesis was discarded. The most important finding of the present study was that the meniscus prosthesis in its present form caused mechanical problems like knee stiffness and implant tears. The meniscus prosthesis and its surgical procedure did not have a negative impact on the patients’ knees when the implants were removed: the clinical scores after explantation were comparable to the preoperative scores. After several months, the state of the drill holes in the tibia also allowed the placement of a unicondylar knee arthroplasty in two patients without any problems.

Thus, the meniscus prosthesis and the screw fixation provide a stiffer construct than what is present in the native meniscus. This makes a good fit of the prosthesis even more important because it is more difficult to adjust. For this, the pre-planning tool and specially designed trial sizers were introduced. Nevertheless, it proved difficult to achieve a good fit with these 2 instruments. Given the variable morphology of the knees in different patients, multiple adjustment options in the design are needed to make it fit for different patients. These possibilities were lacking in this design of the meniscus prosthesis. Once the location of the screws and the size of the prosthesis were determined, there were no more escapes and the fit could only rely on adjustments due to creep. This may have been insufficient in the majority of patients, and consequently may have played a role in the failure of the prosthesis.

This artificial meniscus prosthesis is the first anatomic artificial meniscus that has been placed in a patient. The prosthesis is a non-resorbable solid implant which does not require the ingrowth of tissue. It is made of PCU and is flexible in compression but stiff in tension due to its softer outer layer and a reinforcement ring in the core of the implant. It is not clear how much force passes through the native meniscus during walking, but it shows elastic moduli of between 60 and 160 MPa in a circumferential direction, allowed by the longitudinal orientation of the collagen fibres [5]. To approach the circumferential stiffness of the native meniscus, a reinforcement ring was introduced. However, the ring also increased the stiffness in the horizontal plane due to the flat geometry of this reinforcement ring and made the prosthesis less able to adjust than the native meniscus to the changing geometry of the femoral condyle and its translation on the tibia plateau during flexion and extension of the knee. This may have impaired the recovery of knee mobility after implantation.

The rigid construction of the implant may also explain why all patients (initially) experienced a restricted range of motion and some effusion. The stiff design, together with the rather rigid fixation with screws, made the artificial meniscus prosthesis less mobile than the native meniscus.

Only 1 patient reached the 2-year follow-up visit with an intact prosthesis. One year after surgery, knee function began to improve and scores increased. Why the prosthesis survived specifically in this patient remains unclear. Grammens et al. described the different morphotypes of the medial compartment and their relation to (meniscus) degeneration [4]. The implant geometry may have fit better in this patient: the specific geometry of the knee in this patient may have led to a less destructive loading of the prosthesis and, consequently, to a longer survival. The meniscus prosthesis is still in situ in this patient and follow-up will be continued.

For several other orthopaedic procedures a virtual sizing tool is a valuable addition to the pre-operative planning. In this study this tool gave the orthopaedic surgeons the opportunity to virtually place the prosthesis on 3D reconstructions of the patients’ knees and estimate the size of the prosthesis and its position on the tibial plateau. In 4 out of 5 patients, however, there was a size difference between what was predicted and what was ultimately placed. Three times a smaller size was placed, once a larger size. It is unknown why this mismatch occurred and whether it influenced the failure of the implants. The number of patients is too small to investigate causal relationships and more experience will have to be gained with this tool to determine the added value.

One patient reached the 2-year follow-up period and reported significantly less pain after the procedure. Also, the other patients anecdotally reported that pain levels decreased after the implantation of the meniscus prosthesis. This is in line with the experience with other meniscus tissue replacement implants (allografts [7], scaffolds [10] and artificial permanent implants [12]) that pain in the affected compartment decreases when resected meniscus tissue is replaced with another material. Although several groups report meniscus allograft extrusion from the joint and scaffold shrinkage, the clinical results are still good [1]. This may imply that a small improvement in load distribution, change in shear forces or added stability may have a beneficial effect on the pain in the affected knee compartment.

This trial was the first effort to replace the resected medial meniscus with this anatomic artificial meniscus prosthesis. The failure mechanisms provided input for the improvement of the next version of the meniscus prosthesis. Although only 1 patient reached the 2-year follow-up visit, this patient showed significant improvement in PROMs and function of the knee, which suggests that these implants have the potential to contribute to the treatment of post-meniscectomy pain syndrome. The clinical findings described in this first-in-human trial are instrumental in the process of developing a meniscus prosthesis that significantly improves the daily lives of patients with post-meniscectomy pain syndrome and provide crucial input to the next implant design and clinical procedure.

In terms of clinical relevance, this study confirms how difficult it is to develop a meniscus prosthesis that can resist the huge forces in the knee joint. The results from this study may be of importance for other groups that are also developing an artificial meniscus.

The results of the current study should be interpreted in the context of its limitations. The number of patients that have received the meniscus prosthesis is low. Therefore, it is impossible to draw any conclusions about the clinical effect of this procedure. A failure cause analysis on only 4 explanted prostheses is also limited but it was unethical to operate on more patients.

## Conclusion

This first version of a meniscus prosthesis led to impaired knee function and breakage of the implant in 4 out of 5 patients. The next version needs more flexibility to accommodate different knee geometries. Nevertheless, the results in the patient still in the study are promising and these results are hopeful for a good solution for this patient category. In the patients in whom the prosthesis was removed, no extra damage was observed compared to pre-implantation and the symptoms returned to pre-study levels.
